# Tailoring Synthetic Pelargonic Acid Esters for Bio-Based Lubricant Applications: Exploring the Relationship between Structure and Properties

**DOI:** 10.1021/acssuschemeng.3c02882

**Published:** 2023-08-10

**Authors:** Michele Emanuele Fortunato, Francesco Taddeo, Rosa Vitiello, Rosa Turco, Riccardo Tesser, Vincenzo Russo, Martino Di Serio

**Affiliations:** Department of Chemical Sciences, University of Naples Federico II, via Cintia, Napoli 80126, Italy

**Keywords:** pelargonic acid, fatty acid alkyl esters, esterification, structure−properties relationship, bio-based lubricants

## Abstract

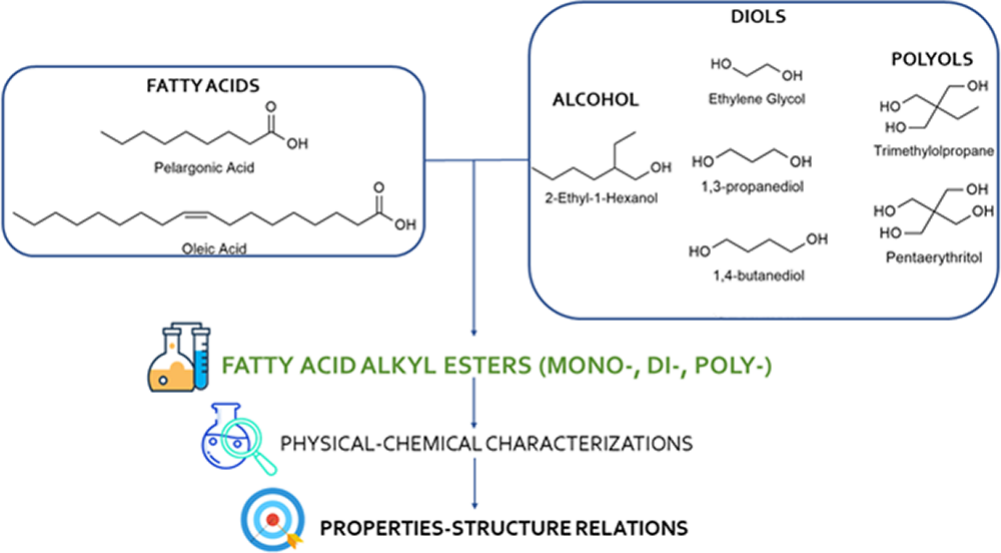

Pelargonic acid (PA) is commercially obtained by oxidative cleavage of fatty acid double bonds. Its esters are interesting compounds used to create bio-based products. An industrially relevant application of these compounds is in the field of solvent manufacturing and formulation of green lubricating oils. The physical–chemical and rheological properties of these esters are influenced by the structural features of the alcohol used as starting materials, such as chain length, number of unsaturation, and degree of branching. This work provides an in-depth study of the existing structure–properties relations for fatty acid alkyl esters obtained from PA and different alcohols [i.e., 2-ethylhexanol (EtHex), ethylene glycol, 1,3-propanediol, 1,4-butanediol, trimethylolpropane, and pentaerythritol]. The aim is to evaluate the use of the synthesized product for the formulation of bio-based lubricants. The chosen alcohols are frequently employed in the preparation of bio-based lubricants. In addition, most of them, such as EtHex and diols, can be derived from biomass sources, contributing to the sustainability of the obtained products. For comparison purposes, some of these alcohols were also used for the synthesis of the corresponding oleic acid esters, which were chosen as a benchmark due to their common use in the synthesis of bio-based lubricants. The influence of the structural factors on the viscosity, pour point (PP), and oxidation stability of the synthesized esters was highlighted by comparing the obtained results. Pelargonates showed lower viscosities and higher PPs than that of the oleates, but they present high stabilities to the oxidation due to the absence of unsaturation.

## Abstract 2

The study of pelargonic acid ester’s properties contributes to the development of new sustainable resources for bio-based products manufacturing.

## Introduction

The depletion of fossil fuels and the growing awareness of the harmful effect of petroleum-based goods on the environment have highlighted the need of using bio-based feedstock to produce chemicals.^1^ The exigency of finding an alternative to petroleum-based raw materials is accentuated by the unpredictability of the petroleum supply (at this historical moment more than ever before) which is heavily impacted by the rising global geopolitical risk over the last 30 years. Research^2,3^ indicates that geopolitical uncertainty leads to long-term volatility in oil prices. Conflicts and wars between oil-importing and oil-producing nations contribute to this risk, with Europe particularly vulnerable due to its reliance on oil imports.^4^ Therefore, embracing bio-based feedstock and reducing dependence on petroleum not only aligns with environmental concerns but also mitigates risks associated with geopolitical factors, promoting a more sustainable and resilient chemical industry. Bio-based raw materials are derived from biomass sources, such as plants, algae, animal, and vegetable wastes. Their use can direct chemical industries toward a more sustainable and circular economy.

Fatty acids are among the most important chemicals obtained from renewable sources since their derivatives, such as fatty acid alkyl esters (FAAEs), find different applications. In this regard, there is an increasing interest in the industrial use of pelargonic acid (PA). This saturated nine-carbon fatty acid is naturally occurring in various vegetables, fruits, such as oranges, grapes, and apples, and in animal fats.^5^ It is industrially produced through oxidative cleavage of oleic and erucic acid, which are sourced from edible, non-edible, and waste vegetable oils, together with azelaic acid from which it is separated through fractional distillation.^6−8^ Additionally, PA can be extracted to a lesser extent from edible plants such as *Pelargonium*.^5,9^ Pelargonic acid esters (PAEs) are extremely versatile products that can be used as solvents, plasticizers, intermediate for cosmetic and pharmaceuticals formulations, and as additives for biofuels. As an example, it has been reported^10^ that 2-ethylhexylpelargonate showed excellent properties as a solvent for printing ink formulations. In addition, it is known that polyol esters of the PA (i.e., trimethylolpropane tripelargonate (TMPTP) and pentaerythritol tetrapelargonate (Pe)] can be used as lubricant additives being one of the main components of jet engine lubricating oils.^11^

Worthy of note is the use of FAAEs as bio-based lubricants, which enjoy the properties of sustainability because they are obtained from renewable sources, biodegradability, low ecotoxicity, and do not contribute to Volatile Organic Compounds emission.^12−14^ Their better lubricity than mineral-based oils is related to their ester functionality.^15^ In fact, the polarity of these molecules favors the interaction with the metallic surface resulting in the formation of a high-strength lubricating layer that reduces friction and wears.^16^

Oleic acid (OA) is most frequently used to produce bio-based lubricants due to its huge abundance in vegetable oils, non-edible oil, and in animal tissues.^17^ Esters of OA, obtained through the esterification of the latter with diols or polyols, such as neopentyl glycol, trimethylolpropane (TMP),^18−20^ and Pe,^21^ are widely adopted to produce green lubricating oils with good rheological and tribological properties. They are used as gear oils, hydraulic oils, compressors, pump oils, and turbine oils,^22^ but the presence of unsaturation leads to scarce thermal and oxidation stabilities preventing their use at high temperatures in a closed system of lubrication, such as internal combustion engines. On the contrary, PAEs are completely resistant to oxidation due to the absence of double bonds on the fatty acid backbone of these molecules, so they can maintain their lubricating properties for longer time when exposed to high temperature and oxygen. It is clear that the physical–chemical, thermal, and flow properties of these esters are strictly dependent on the chemical structure of the carboxylic acid and the alcohol they are derived from.

In this study, different PAEs were obtained through the esterification of PA with a monofunctional alcohol [2-ethylhexanol (EtHex)], diols [ethylene glycol (EG), 1,3-propanediol (1,3-PD), 1,4-butanediol (1,4-BD)], and polyols (TMP, Pe) that are commonly used in the preparation of green lubricating oils.^23^ In addition, EtHex^24,25^ and diols^26^ can be easily derived from biomass sources, whereas polyols are mainly produced from petroleum-based raw materials. Despite the industrial interest for these products, there are no papers in the literature that deal with the structure–properties relations of pelargonates. Furthermore, OA esters were synthetized via the esterification of OA with one representative alcohol from each class (i.e., EtHex, 1,4-BD, TMP, and Pe) and characterized to compare the results. Oleates were synthesized as reference compounds, given their widespread use in the synthesis of bio-based lubricants.

The aim of the present study is to critically evaluate how the final product’s chemical and physical properties, namely, dynamic viscosity, pour point (PP), and oxidation stability, are affected by structural factors such as fatty acid chain length, branching degree, and the presence of unsaturation in order to evaluate their potential use as a base for bio-based lubricants. These properties are of primary importance in the field of lubricants, making them critical factors in assessing the potential utilization of these esters in lubrication. The vast array of the products that were analyzed and the collection of the results that are presented in this paper can be an effective tool for determining the ideal combination of fatty acid and alcohol to produce a synthetic ester with specific properties for the various applications in the field of bio-based lubricants that might be possible.

### Experimental Section

#### Materials

PA (>96 wt %), Pe (98 wt %), EG (>99 wt %), and zinc oxide (99.9 wt %) were purchased from Sigma Aldrich. 1,4-BD (99 wt %), 1,3-PD (99 wt %), EtHex (99 wt %) were obtained from Alfa Aesar. TMP (>98 wt %) was sourced from Fluka Chemicals. OA was supplied by Alfa Aesar (Technical grade, 92 wt %).

#### Characterization of the Reagents and Products

##### Acidity Number, Iodine Value, and Saponification Number

The acidity number (AN) was determined by following the standard method ASTM D664. The conversion *x*_FA_ (%) of the fatty acid was calculated from the initial acidity number AN_i_ and the AN at the end of the reaction AN_f_ according to eq 1:
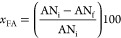
1

The iodine value (IV) provides an estimation of the relative degree of unsaturation of an oil component (in this case that of synthesized FAAEs) as determined by the uptake of iodine. IVs of the products were calculated by following the test method ISO 3961.

The saponification number (SN) indicates how much potassium hydroxide is needed to saponify 1 g of oil, and it is useful to evaluate the content of ester linkages. The higher the SN the more short- and medium-chain fatty acids are contained in the oil sample. The SN of the FAAEs was determined by following the test method NOM 163 07/2003.

##### Oxidation Stability

According to ASTM D8206, the oxidation stability of the FAAEs was assessed using an accelerated aging method on an oxidation stability tester (Anton Paar RapidOxy100). The test was conducted with 5 mL of the sample in the oxidation chamber at a temperature of 120 °C and a maximum pure oxygen pressure of 920 kPa. The oxidation stability is expressed as the time needed by the sample to consume 10% of the oxygen in the oxidation chamber. It has been shown that this rapid oxidation test can be considered an alternative to the Oil Stability Index Analysis method, which has excellent reliability in characterizing the oxidation stability of oils.^27,28^

##### Pour Point

The PPs of the synthesized esters were measured by using an Anton Paar Cold Filter Plugging Point Tester (Model Callisto 100) according to ASTM D 97. The sample was put in a glass test jar and cooled to the temperature at which the liquid does not show any movement inside the jar held in a horizontal position for 5 s.

##### Dynamic Viscosity

The measurements of the dynamic viscosity (η) were performed by using an Anton Paar Rheometer MCR 92 according to the DIN EN 14770 method. The values of dynamic viscosity were obtained, for all the samples, at temperatures ranging from 20 to 100 °C by using a concentric cylinder geometry measuring system. The analyses were carried out using shear rates varying from 1 to 1000 s^–1^.

##### Fourier Transform Infrared (FT-IR)

The products were analyzed by a Nicolet Avatar 360 spectrophotometer. The spectrum was obtained on 4000–600 cm^–1^ for 32 repeated scans using a KBr crystal window (diameter × thickness equal to 25 × 2 mm^2^).

##### ^1^H-NMR

The ^1^H-NMR spectra were obtained by using a Bruker Avance Ultrashield (Bruker Corporation, Billerica, MA, USA) operating at a proton frequency of 400 MHz. The adopted solvent was CDCl_3_. ^1^H-NMR analysis was also used to determine the saturated fatty acids, linoleic acid, and linolenic acid content of the two OAs of different purity by following a method described by Popescu et al.^29^

#### Esterification Reactions

The esterification reactions of PA and OA with six different alcohols, i.e., EtHex, EG, 1,3-PD, 1,4-BD, TMP, and Pe, were carried out in the presence of ZnO as the catalyst,^30^ under the same experimental conditions, fixing the reaction temperature at *T* = 180 °C, the reaction time at *t* = 18 h, the catalyst amount at 2 mol % referred to FA, a FA loading of 150 g, and a FA/alcohol molar ratio of 1 (based on the alcohol’s hydroxyl functional groups). The mass of alcohol was calculated as is shown in eq 2:
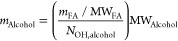
2

The experimental procedure can be summarized as follows: the fatty acid (150 g, 0.950 mol for PA, and 0.531 mol for OA) together with the alcohol were added to a 500 mL Schlenk flask. Then, the ZnO catalyst (1.546 g, 0.019 mol for PA esterification; 0.864 g, 0.011 mol for OA esterification) was added to the mixture. The reactants were mixed through a magnetic stirrer and heated to 180 °C by using an oil bath. Vacuum distillation (*p* = 50 mbar) was used to remove the water that was produced during the reaction for the first 9 h, followed by nitrogen stripping for the remaining time, to reach a conversion ≥98% calculated as in eq 1 (see the Characterization of the Reagents and Products section). At the end of the reaction, the system was allowed to cool overnight to room temperature and the precipitated catalyst was recovered by filtration and centrifugation (3000 rpm for 30 min; for oleates this step was repeated twice). The experimental setup used for the synthesis of FAAEs is shown in Figure S1.

#### Statistical Tools

For each analysis, described in the previous paragraphs, *n* = 3 measurements were carried out on the same sample to evaluate the standard deviation (σ). In addition, the synthesis of the alkyl esters was repeated twice, and the products were reanalyzed to verify the repeatability of the results. The results are reported as the mean value *±*2σ by assuming a confidence interval of ±95%.

## Results and Discussion

The chemical composition of the technical grade OA was determined by ^1^H-NMR analysis. The results are reported in Table 1 together with the ANs and the IVs.

**Table 1:** Chemical Composition and Physico-Chemical Properties of the Technical Grade Oleic Acid (Product Number: A16663)

composition	value
oleic acid [mol%]	92.51
linoleic acid [mol%]	3.43
linolenic acid [mol%]	0.11
saturated FA [mol%]	3.95
chemical–physical property	
acidity number [mg_KOH_/g]	207 ± 1
iodine value [g_I2_/100 g]	85 ± 3

The synthesized esters were subjected to chemical–physical characterization. The set of the results for the PA esters, namely, 2-ethylhexyl pelargonate (EtHexP), ethylene glycol dipelargonate (EGDP), 1,3-propanediol dipelargonate (1,3-PDDP), 1,4-butanediol dipelargonate (1,4-BDDP), TMPTP, and PeTP, and oleates, i.e., 2-ethylhexyl oleate (EtHexO), 1,4-butanediol dioleate (1,4-BDDO), trimethylolpropane trioleate (TMPTO), and pentaerythritol tetraoleate (PeTO) are listed in Tables 2 and 3, respectively.

**Table 2:** Results of the Analysis Conducted on the Pelargonic Acid Esters^a^

PAEs	CAS number	purity [%]	AN [mg_KOH_/g]	SN [mg_KOH_/g]	ox. stab. [min]	PP [°C]	η@40 °C [mPa s]	η@100 °C [mPa s]
EtHexP	59587-44-9	99.8	2.7 ± 0.1	210 ± 10 (207^b^)	>300	<−45 ± 1	4.1 ± 0.1	2.1 ± 0.1
EGDP		95.1	0.6 ± 0.1	340 ± 20 (327^b^)	>300	9* ± 1	6.6 ± 0.1	2.8 ± 0.1
1,3-PDDP	28267-33-6	96.0	1.0 ± 0.1	300 ± 10 (314^b^)	>300	9* ± 1	7 ± 1	3.0 ± 0.1
1,4-BDDP	16805-92-2	97.8	2.2 ± 0.1	280 ± 10 (303^b^)	>300	9* ± 1	9 ± 1	3.3 ± 0.1
TMPTP	126-578	93.4	0.9 ± 0.1	300 ± 10 (303^b^)	>300	–36 ± 1 (−51^c^)	21.5 ± 0.4 (21^c^)	5 ± 1 (4.6^c^)
PeTP	14450-05-06	94.1	0.7 ± 0.1	350 ± 20 (322^b^)	>300	–3 ± 1 (−7^c^)	32 ± 1 (32^c^)	6 ± 1 (6^c^)

a Values marked by * are referred to as esters that are completely solid at 6 °C.

b Calculated value.

c Boyde.^23^

**Table 3:** Results of the Analysis Conducted on the 92% Pure Oleic Acid Esters

OAEs	CAS number	purity [%]	AN [mg_KOH_/g]	ox. stab. [min]	PP [°C]	η@40 °C [mPa s]	η@100 °C [mPa s]
EtHexO	26399-02-0	96.0	1.0 ± 0.1	46 ± 1	–36 ± 1 (−36^c^)	8.3 ± 0.2 (9.65^d^)	3.2 ± 0.1
1,4-BDDO	39903-07-6	99.0	1.3 ± 0.1	52 ± 2	+6 ± 1	29 ± 1 (21^b^)	7.4 ± 0.3 (6.2^b^)
TMPTO	57675-44-2	94.7	1.4 ± 0.1	45 ± 2	–37 ± 1 (−39^a^)	56 ± 1 (46.8^a^)	10.6 ± 0.2 (9.4^a^)
PeTO	242-960-5	94.8	2.1 ± 0.1	50 ± 2	–21 ± 1 (−21^a^)	72 ± 2 (64^a^)	13 ± 1 (10^a^)

a Boyde.^23^

b Raghunan.^31^

c Asadauskas.^32^

d Kai.^33^

The theoretically calculated SNs and the values of the other parameters, found in the literature, are reported in the same tables for comparison. To ensure easy readability of the tables for the reader and due to space constraints, only the viscosity values calculated at 40 and 100 °C (since they are commonly used as reference temperatures for the viscosity of lubricants) are presented in the tables.

EGDP was never synthesized in the previous studies reported in the scientific and patent literature. All the other products were already synthetized, being classified with a CAS number; however, only EtHexP, 1,3-PDDO, TMPTO, and PeTO are REACH registered compounds. For EtHexP, EGDP, 1,3-PDDP, 1,4-BDDP, the authors found no data on viscosity, PP, or oxidative stability in the literature.

The ^1^H-NMR spectra confirmed the structures of the FAAEs. The ^1^H-NMR technique was employed to determine the purity of the synthesized esters (Tables 2 and 3). Further details regarding the purity calculation methods can be found in the Supplementary Material. In particular, for the novel compound, ^13^C-NMR analysis was conducted confirming further the purity of the synthesized product (see the Supplementary Material). As an example, Figure S10 shows the ^1^H-NMR and FT-IR spectra of the synthesized TMPTO, respectively. The singlet peak at 4.03 ppm is attributed to the methylene groups of the TMP backbone bonded to the carboxylate groups of the fatty acid chains (−CH_2_OC(*O*)R) related to the triester. To support this, FT-IR spectrum shows typical intense peaks of an ester: one at 1740 cm^–1^, due to −C=O stretching vibration, and the other at 1163 cm^–1^ related to the C–O–C single bond stretching vibration; furthermore the −OH peak at ∼3500 cm^–1^ is very low indicating the presence of small amount of unreacted carboxylic acid and/or alcohol.

### Trend of the Dynamic Viscosities

**Figure 1:**
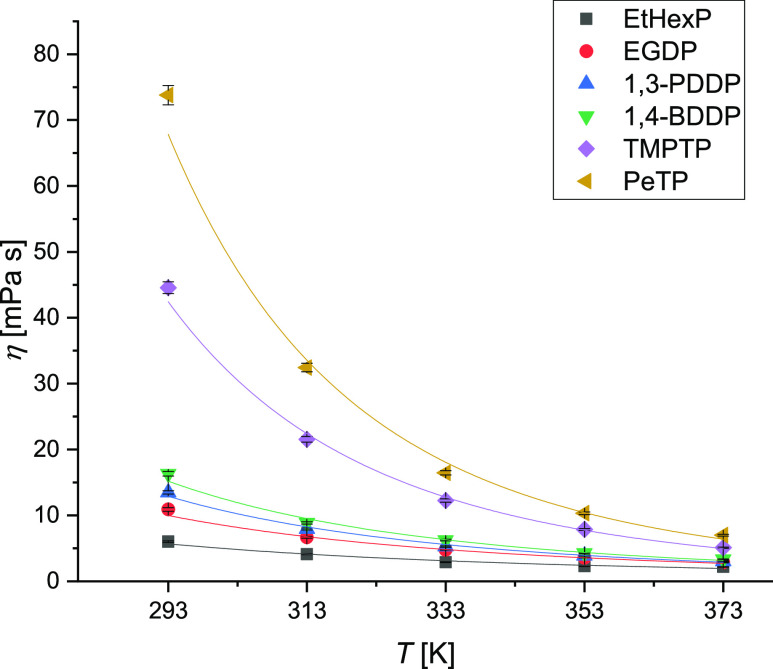
Viscosity trend with temperature of pelargonic acid esters.

**Figure 2:**
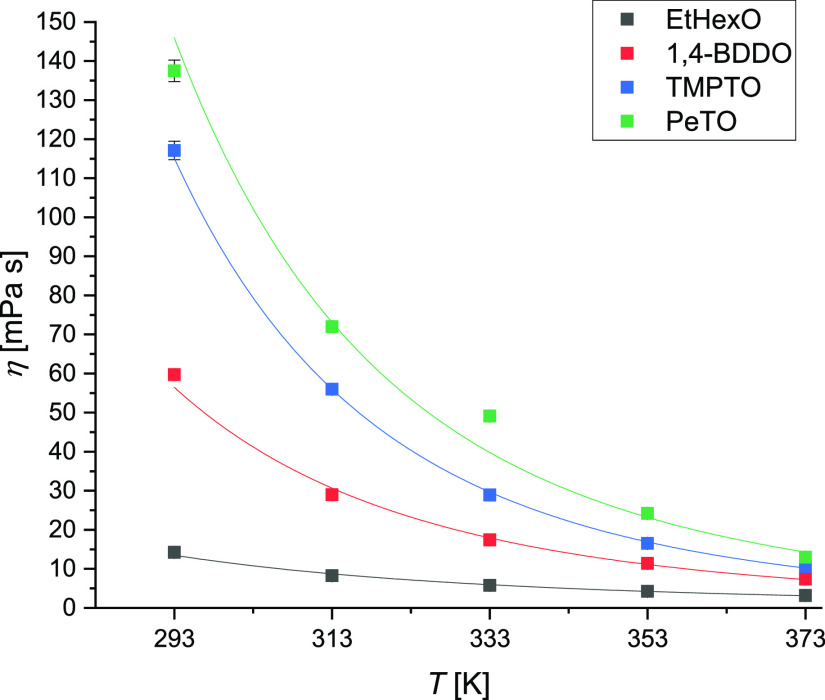
Viscosity trend with temperature of oleic acid esters.

**Figure 3:**
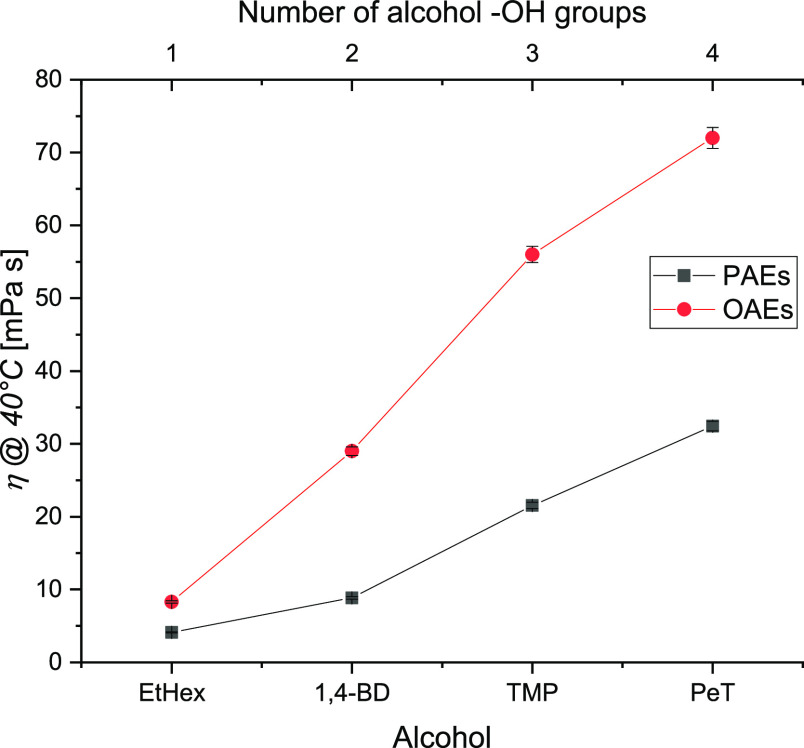
Effect of the number of alcohol −OH groups on the viscosities of pelargonates and oleates.

Viscosity is one of the main properties that influences the applicability of FAAEs as bio-based lubricants. Higher viscosities result in a higher fluids’ resistance to flow, which also affects its ability to form a lubricating film.^34^ On a molecular level, a higher degree of random intermolecular interactions and a reduced flexibility of the molecules has a favorable effect on the viscosity of an ester. Flexibility is reduced by introducing branching, while an increase of the intermolecular interactions is obtained by increasing the carbon chain length of the raw materials or enhancing the raw material functionality (more ester groups per molecule) resulting in higher molecular weights.^23^ The values of viscosity of pelargonates and oleates at different temperatures (from 20 to 100 °C) are shown in Figures 1 and 2, respectively. As obvious, the dynamic viscosities of OAEs are higher than that of the correspondent PAEs. The viscosity of PA diesters (i.e., EGDP, 1,3-PDDP, and 1,4-BDDP) increases with the length of the alcoholic segment in the ester molecule (Figure 1). The dynamic viscosity rises by passing from monoesters to polyol esters as the number of alcohol functional groups increases. This is valid for both pelargonates and oleates at all the investigated temperatures. Figure 3 shows the effect of the number of −OH functional groups on the values of viscosity of PAEs and OAEs at *T* = 40 °C. In general, the differences in viscosity values between the esters are more pronounced at lower temperatures than at higher, particularly for the polyol esters. It is important to note that different structural impacts on the values of dynamic viscosity are generally more pronounced at lower temperatures where molecular mobility is significantly more constrained, and they become less pronounced at higher temperatures.^35^

The viscosity dependence on temperature follows an Arrhenius-like law:^36^
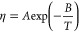
3where *A* and *B* are the parameters, and *T* is the temperature (K). The agreement between the measured data and the fitting lines in Figures 1 and 2 confirms the validity of the proposed exponential law, which is supported by coefficients of determination *R*^2^ values greater than 95%. The estimated values of *A* and *B* parameters for pelargonates and oleates are reported in Table 4. The exponential parameter (*B*) is a measure of how much viscosity varies with temperature. Higher absolute values of *B* indicate greater changes in viscosity with temperature variations. Among the synthetized esters, polyol esters of pelargonic and OA (specifically TMPTP, PeTP, TMPTO, and PeTO) exhibit the highest *B* values (as shown in Table 4) and consequently the most significant reduction in viscosity as the temperature increases. For instance, PeTP experiences a viscosity decrease of 90% when passing from 20 to 100 °C, while EGDP’s viscosity only decreases by 70%. It has been documented in the literature^23^ that esters incorporating inflexible structural elements, such as branching, demonstrate a higher sensitivity of viscosity to temperature fluctuations.

**Table 4:** Estimated Parameters for the Viscosity Dependencies with Temperature

pelargonates	*A* [mPa s]	relative error [%]	*B* [K]	relative error [%]
EtHexP	0.03745	15	–1471.61	11
EGDP	0.02298	20	–1779.62	5
1,3-PDDP	0.0119	19	–2047.46	4
1,4-BDDP	0.00993	16	–2148.57	5
TMPTP	0.00192	15	–2930.49	2
PeTP	0.00111	25	–3227.73	5

oleates	*A* [mPa s]	relative error [%]	*B* [K]	relative error [%]
EtHexO	0.01476	25	–1998.2	4
1,4-BDDO	0.00402	28	–2798.28	3
TMPTO	0.00144	16	–3307.35	1
PeTO	0.00292	16	–3170.5	7

### Trend of the Pour Point

**Figure 4:**
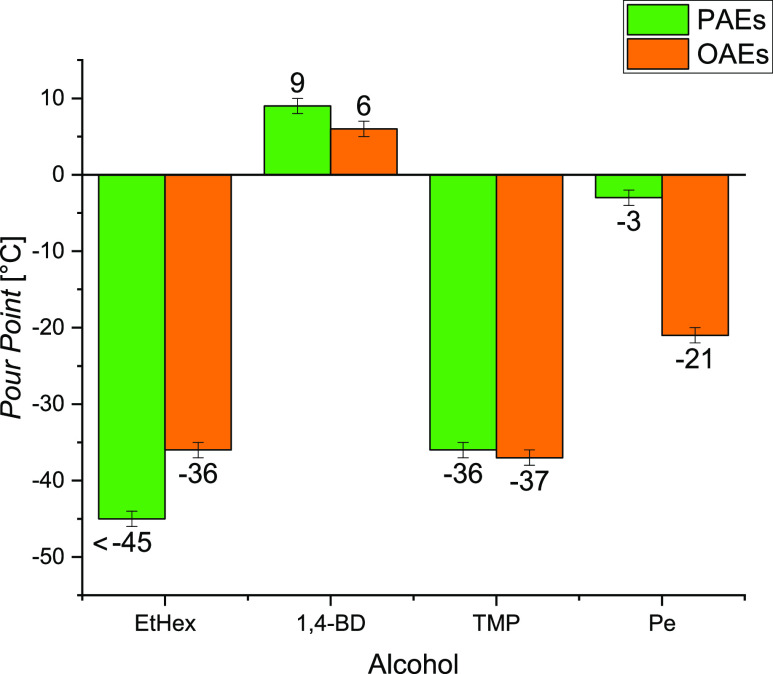
Trend of the pour points of pelargonates and oleates.

The PP, like viscosity, is a structure-sensitive property that is influenced by molecular interactions and molecule packing efficiency.^31,37,38^ The PPs of the PAEs and OA esters are compared in Figure 4. The obtained results for PPs are challenging to predict due to the combined influence of molecular weight and steric hindrance. As can be noticed, pelargonates and oleates obtained from branched alcohols (i.e., EtHexP, EtHexO, TMPTP, TMPTO, and PeTO) show very low PPs (from −24 to <−45 °C). Branching, in fact, lowers the crystallization temperature of esters, thereby compromising their crystal packing ability.^39^ In the case of esters derived from EtHex, the PP is primarily influenced by the molecular weight, resulting in a higher PP for EtHexO. However, for 1,4-BD and TMP esters, the effects of molecular weight and steric hindrance are more complex, leading to a balanced effect, giving place to similar PP values for both pelargonates and oleates. An interesting result arises in the case of PeTP, which stops its flow at a higher temperature (−3 °C) compared to the other branched esters, particularly to PeTO. The intrinsic symmetry of the Pe structure can facilitate the molecular packing at decreasing temperatures, being responsible of the higher PP found in the case of Pe esters (both PeTP and PeTO) with respect to oleates and pelargonates obtained from EtHex and TMP. In particular, the big difference between the PP values of PeTP and PeTO can be attributed to the presence of unsaturation that result in a bent configuration of the OA portion of PeTO preventing the close packing of molecules during cooling.^35,40^ Linear dipelargonates, namely, EGDP, 1,3-PDDP, and 1,4-BDDP, are completely solid at 6 °C (see Table 2). Rodrigues^35^ states that linear esters pack efficiently into crystals showing higher PPs because of enhanced van der Waals attractions. On the other hand, the linear dioleate (1,4-BDDO) shows slightly lower PP than the corresponding pelargonate because of the presence of unsaturation on the fatty acid portion of the ester molecule.

### Trend of the Oxidation Stabilities

Oxidation stability is a measure of the resistance of a substance to the attack of oxidizing agents. Oxidation of results in polymerization into a plastic-like consistency and degradation of the ester. For this reason, it is an undesirable process, especially in the case of lubricant application.

It must be stated that the PAEs showed high oxidation stability (>300 min) due to the absence of unsaturation (Table 2), while in the case of OAEs, the oxidation stability was between 45 and 52 min (Table 3). The presence of unsaturation hurts the oxidation stability: a higher total number of unsaturation and a higher degree of unsaturation of the fatty acid chain led to a higher rate of oxidation and, in the case of investigated OAEs, this is the most important parameter considering the low difference in oxidation stability of the different OAEs obtained with different alcohols. The ester group itself is highly resistant to oxidation, andtherefore, the oxidation reaction rate is linked with the rate of initiation that is influenced by the stability of the C–H bond of substituent hydrocarbon groups of the ester molecule. The strength of the C–H bond adjacent to a C–C double bond, particularly that of a hydrogen attached to the carbon between two double bonds, is low so hydrogen is easily removed. For this reason, as the number of double bonds increases there are more susceptible sites to the H atom abstraction and the oxidation occurs at a faster rate.^41^ The trend of the oxidation stability for the oleates can be found in Supplementary Materials (see Figure S13).

## Conclusions

This work aims to explore the relationship between the structure and properties of FAAEs and provides guidance for selecting the appropriate combination of fatty acid and alcohol for the synthesis of an ester to be used as a potential bio-based lubricant. PAEs were synthesized by esterifying pelargonic acid with six different alcohols, including EtHex, EG, 1,3-PD, 1,4-BD, TMP, and Pe. These alcohols were selected for their differences in chain length, number of branching, and number of −OH functional groups. The physical–chemical properties of the resulting pelargonates were evaluated, including dynamic viscosity, PP, and oxidation stability, and compared to those of the oleates obtained by esterifying OA with the same alcohols and used as benchmark products. The results indicate that the dynamic viscosity of pelargonates increases as the number of −OH functional groups in the alcohol increases but remains lower than that of the oleates. In addition, the viscosity of the esters follows an Arrhenius-like trend with the temperature. Notably, EtHexP and TMPTP are suitable for low-temperature applications due to their very low PPs like the corresponding oleates thanks to their branched structure. Additionally, PAEs are highly resistant to oxidation due to the absence of unsaturation, which can significantly affect, on the other hand, the oxidation stability of OA esters.

## Supporting Information Available

^1^H-NMR and IR spectra for the synthetized esters and a scheme of the experimental setup; for the novel compound (ethylene glycol dipelargonate), ^13^C-NMR spectrum reported; trend of the oxidation stability of the synthetized esters; and determination method of the purity of the fatty acid alkyl esters by ^1^H-NMR (PDF)

## Abbreviations

1,3-PD: 1,3-propanediol
1,3-PDDP: 1,3-propanediol dipelargonate
1,4-BD: 1,4-butanediol
1,4-BDDO: 1,4-butanediol dioleate
1,4-BDDP: 1,4-butanediol dipelargonate
DiPE: dipentaerythritol
EG: ethylene glycol
EGDP: ethylene glycol dipelargonate
EtHex: 2-ethylhexanol
EtHexO: 2-ethylhexyl oleate
EtHexP: 2-ethylhexyl pelargonate
FA: fatty acid
FAAE: fatty acid alkyl ester
NPG: neopenthyl glycol
OA: oleic acid
OAEs: esters from 92% pure oleic acid
PAEs: pelargonic acid esters
Pe: pentraerythritol
PeTO: pentaerythritol tetraoleate
PeTP: pentaerythritol tetrapelargonate
TMP: trimethylolpropane
TMPTO: trimethylolpropane trioleate
TMPTP: trimethilolpropane tripelargonate
**Notation**
*A*: pre-exponential factor [mPa s]
*B*: exponential parameter [K]
AN: acidity number [mg_KOH_/g_sample_]
CT: concentration of the titrant [mol L^–1^]
IV: iodine value [g_I2_/100 g_sample_]
*m*_Alcohol_: alcohol mass [g]
*m*_FA_: fatty acid mass [g]
MW_Alcohol_: alcohol molecular weight [g/mol]
MW_Alcohol_: fatty acid molecular weight [g/mol]
MW_KOH_: molecular weight of potassium hydroxide [g mol^–1^]
*n*: number of measurements
*N*_OH,alcohol_: number of hydroxyl functional group in the alcohol
PP: pour point [°C]
SN: saponification number [mg_KOH_/g_sample_]
*t*: reaction time [h]
*T*: temperature
*V*_0_: volume of the titrant in the blank determination [mL]
*V*_T_: volume of the titrant [mL]
*w*_sample_: weight of the oil sample [g]
*x*_FA_: conversion of the fatty acid [%]
**Greek Letters**
η: dynamic viscosity [mPa s]
σ: standard deviation
