# Supplementary material for: Tailoring Synthetic Pelargonic Acid Esters for Bio-Based Lubricant Applications: Exploring the Relationship between Structure and Properties
Source: ACS Sustain Chem Eng. 2023 Aug 10;11(33):12406–13. doi: 10.1021/acssuschemeng.3c02882 (PMC10445259; doi:10.1021/acssuschemeng.3c02882)
Supplement: Supplementary file 1 — sc3c02882_si_001.pdf [file sc3c02882_si_001.pdf]

Tailoring Synthetic Pelargonic Acid Esters for Specific Applications: Exploring the Relationship Between Structure and Properties

Michele Emanuele Fortunato[1], Francesco Taddeo[1], Rosa Vitiello[1], Rosa Turco[1], Riccardo Tesser[1], Vincenzo Russo[1], Martino Di Serio[1,*]

[1]Department of Chemical Sciences, University of Naples Federico II, via Cintia, IT-80126 Napoli, Italy

*corresponding author: Martino Di Serio (diserio@unina.it)

Supplementary Material

Contents

Page S3 – Figure S.1: Experimental setup.

Page S4 – Figure S.2: a) ^{1}H-NMR and b) FT-IR spectra of 2-ethylhexyl pelargonates.

Page S5 – Figure S.3: a) ^{1}H-NMR and b) FT-IR spectra of ethylene glycol dipelargonate.

Page S6 – Figure S.4: a) ^{1}H-NMR and b) FT-IR spectra of 1,3-propanediol dipelargonate.

Page S7 – Figure S.5: a) ^{1}H-NMR and b) FT-IR spectra of 1,4-butanediol dipelargonate.

Page S8 – Figure S.6: a) ^{1}H-NMR and b) FT-IR spectra of TMP tripelargonate.

Page S9 – Figure S.7: a) ^{1}H-NMR and b) FT-IR spectra of pentaerythritol tetrapelargonate.

Page S10 – Figure S.8: a) ^{1}H-NMR and b) FT-IR spectra of 2-ethylhexyl oleate.

Page S11 – Figure S.9: a) ^{1}H-NMR and b) FT-IR spectra of 1,4-butanediol dioleate.

Page S12 – Figure S.10: a) ^{1}H-NMR and b) FT-IR spectra of TMP trioleate.

Page S13 – Figure S.11: a) ^{1}H-NMR and b) FT-IR spectra of pentaerythritol tetraoleate.

Page S14 – Figure S.12: ^{13}C-NMR spectrum of ethylene glycol dipelargonate. Figure S.13- Trend of the oxidation stabilities of oleates.

Page S15 – Determination of the purity of the fatty acid alkyl esters by ^{1}H-NMR.

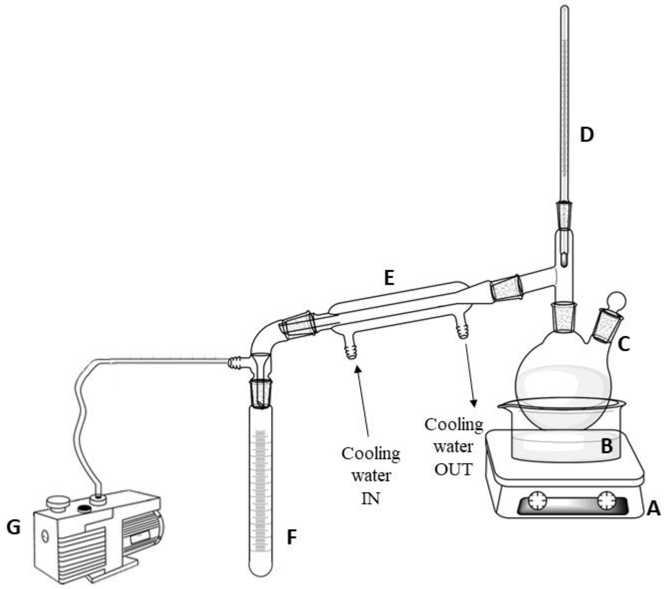

Figure S.1- Experimental setup. A. Heating and stirring plate; B. Oil bath; C. 500 mL Schlenk flask; D. Thermometer; E. Liebig condenser; F. Graduated collector cylinder; G. Vacuum Pump.

a)

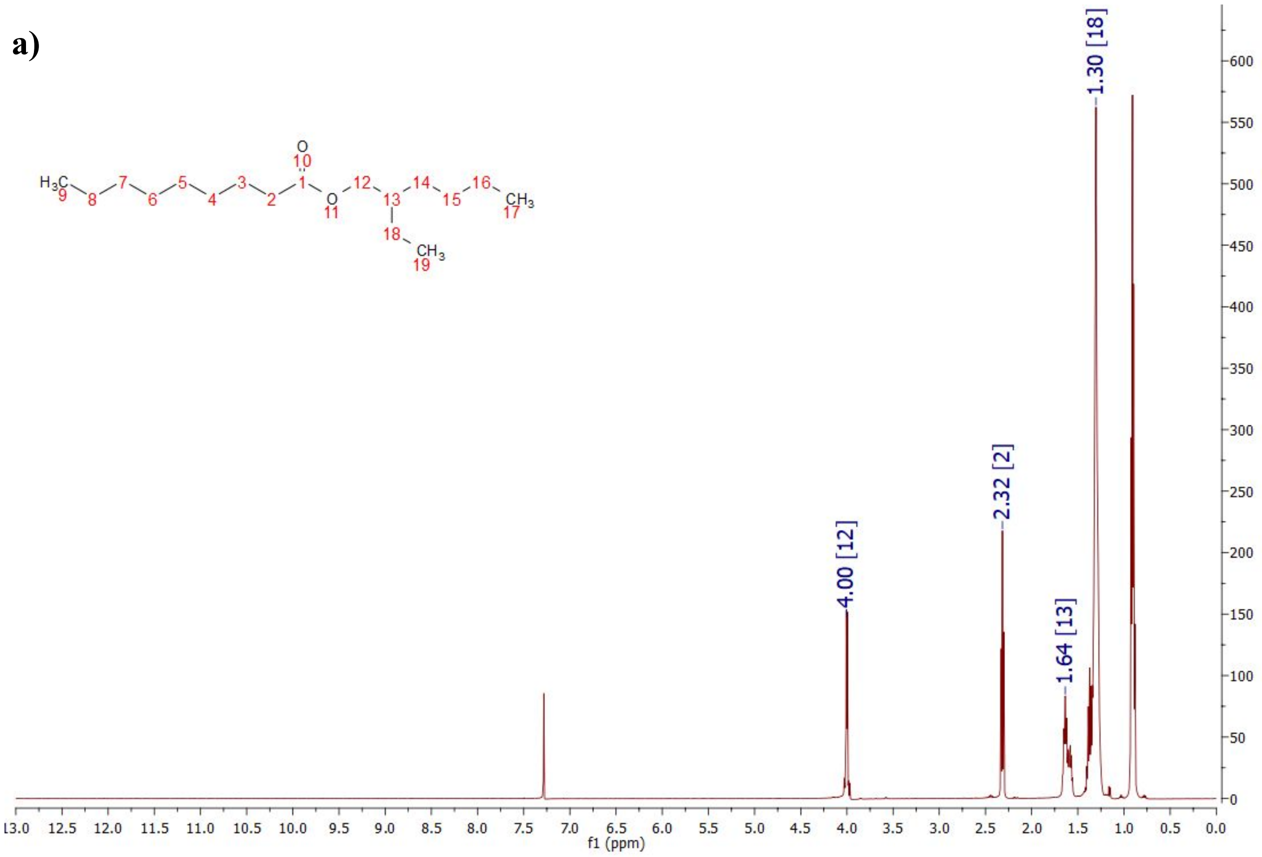

b)

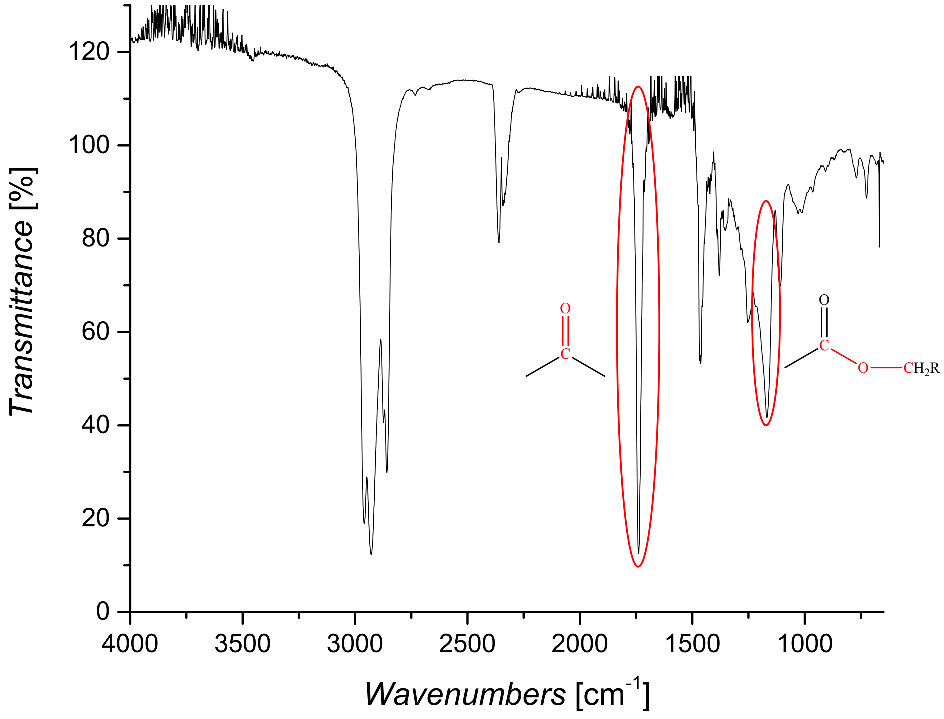

Figure S.2-a) ^{1}H-NMR and b) FT-IR spectra of 2-ethylhexyl pelargonate.

a)

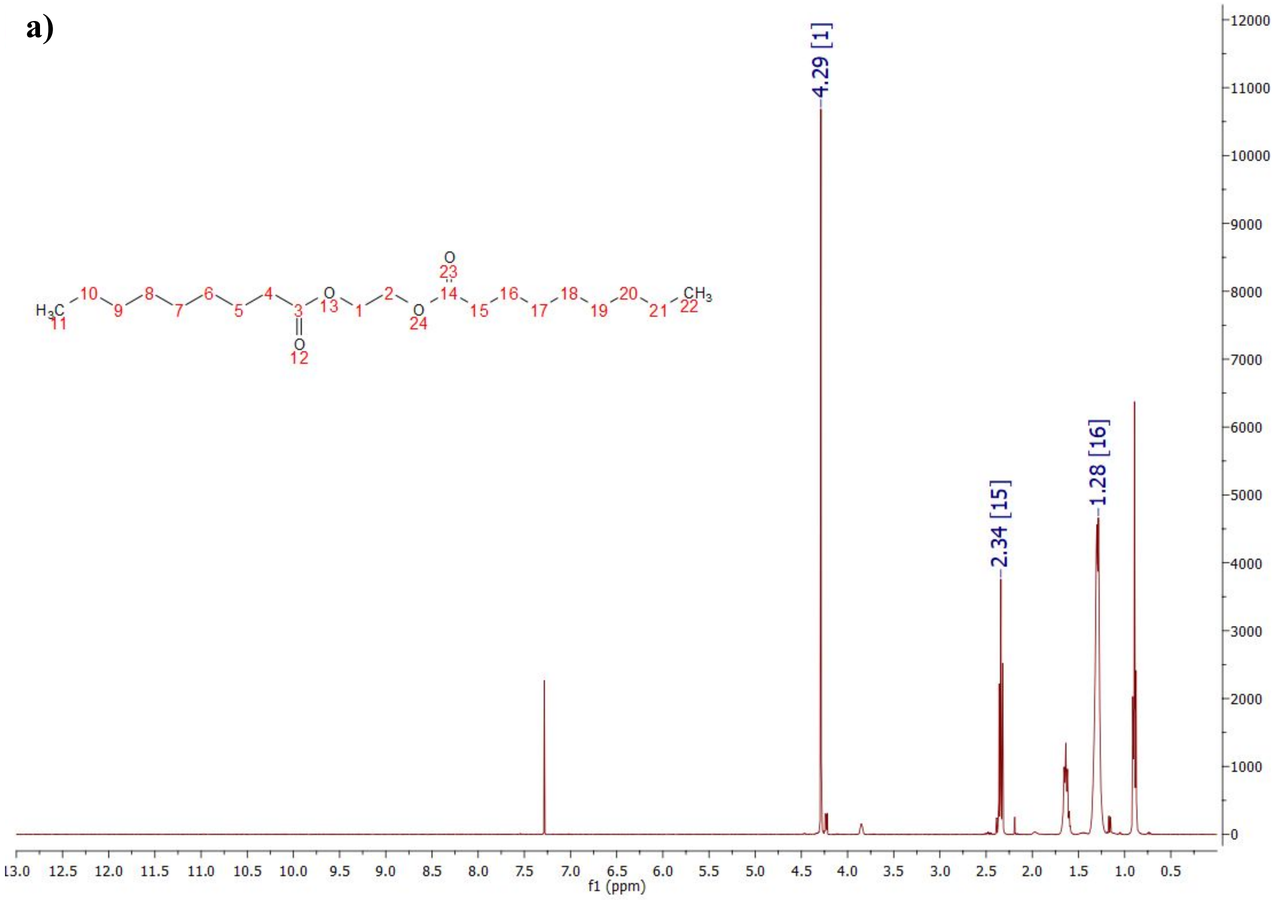

b)

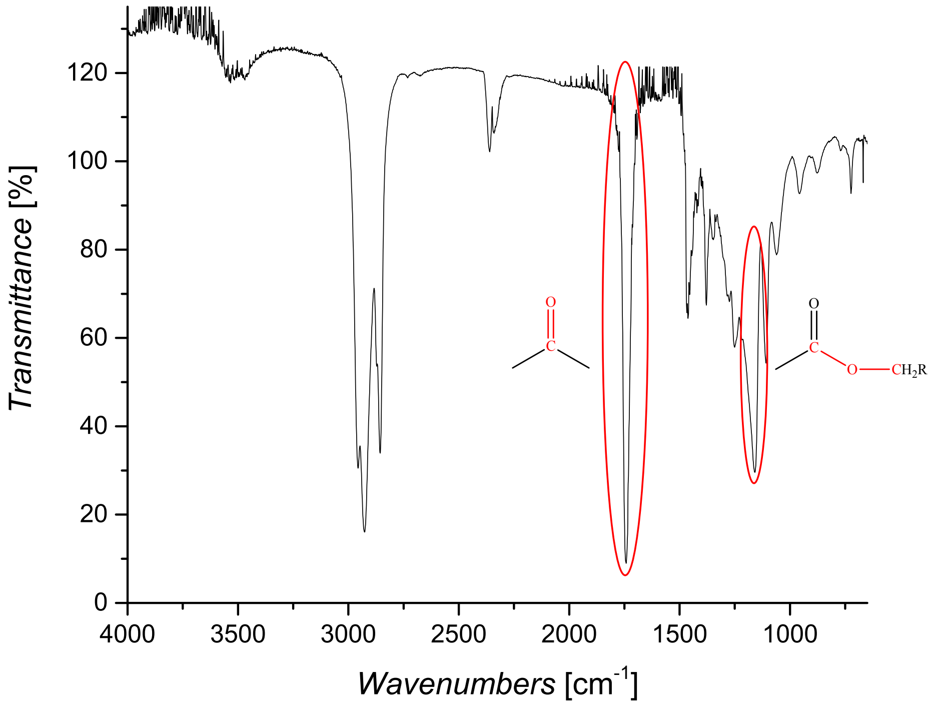

Figure S.3- a) ^{1}H-NMR and b) FT-IR spectra of ethylene glycol dipelargonate.

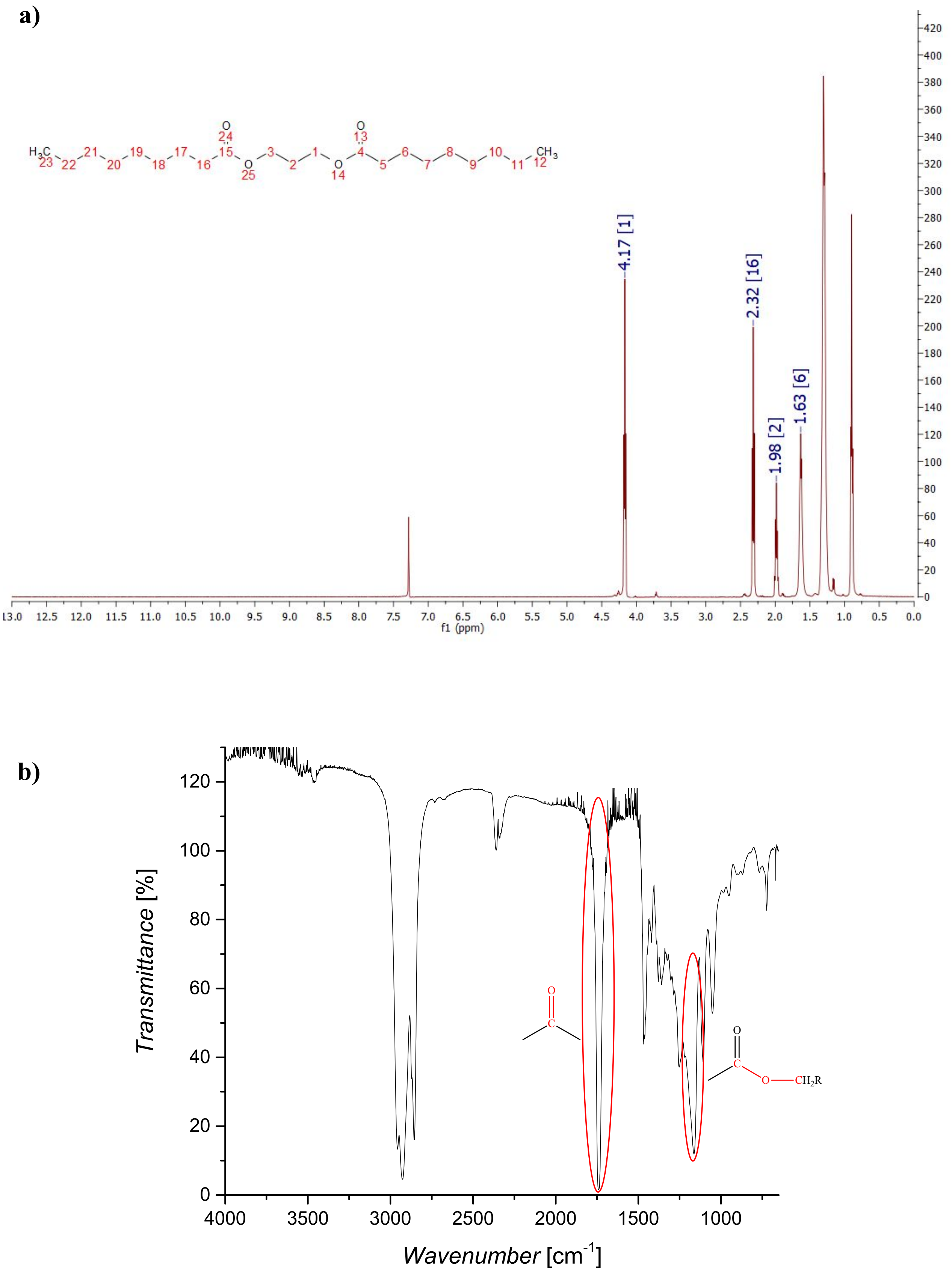

Figure S.4- a) ^{1}H-NMR and b) FT-IR spectra of 1,3-propanediol dipelargonate.

a)

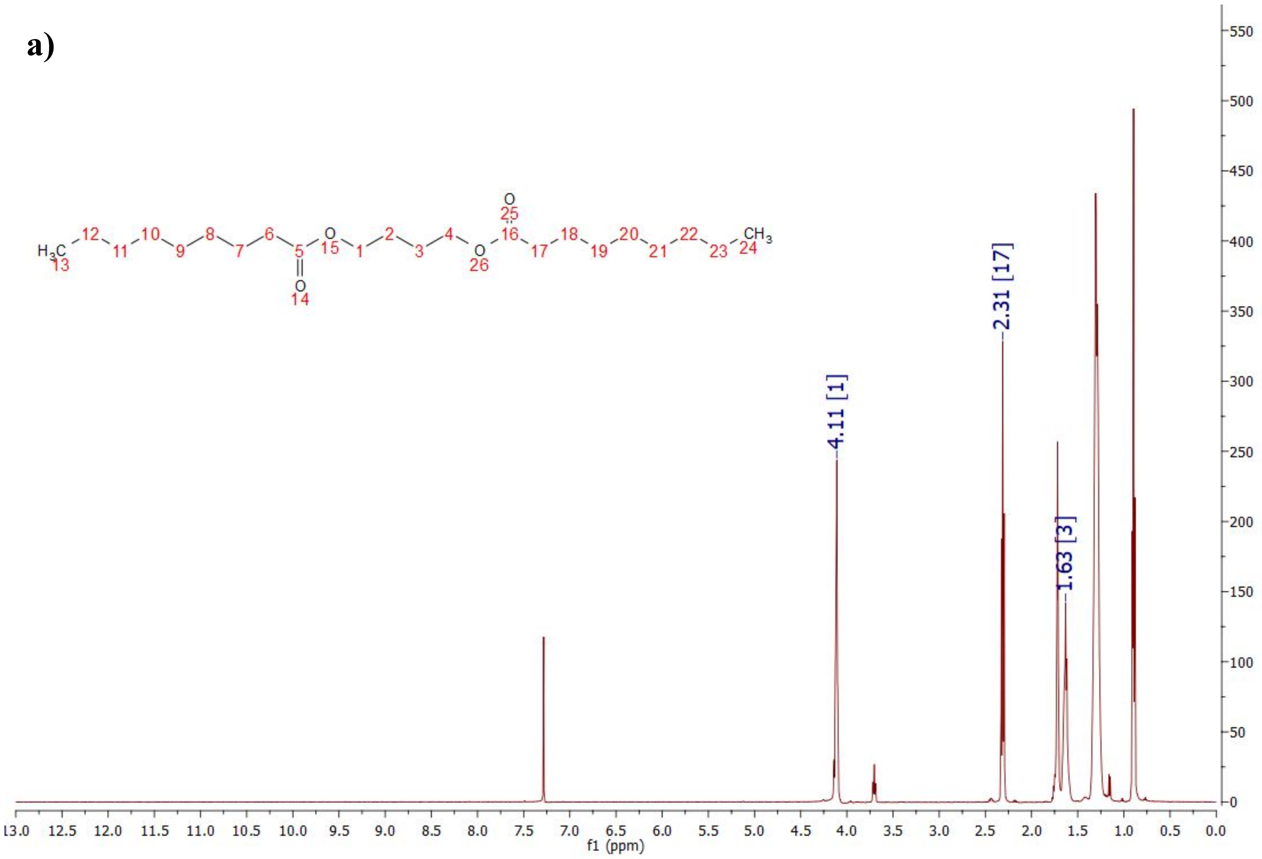

b)

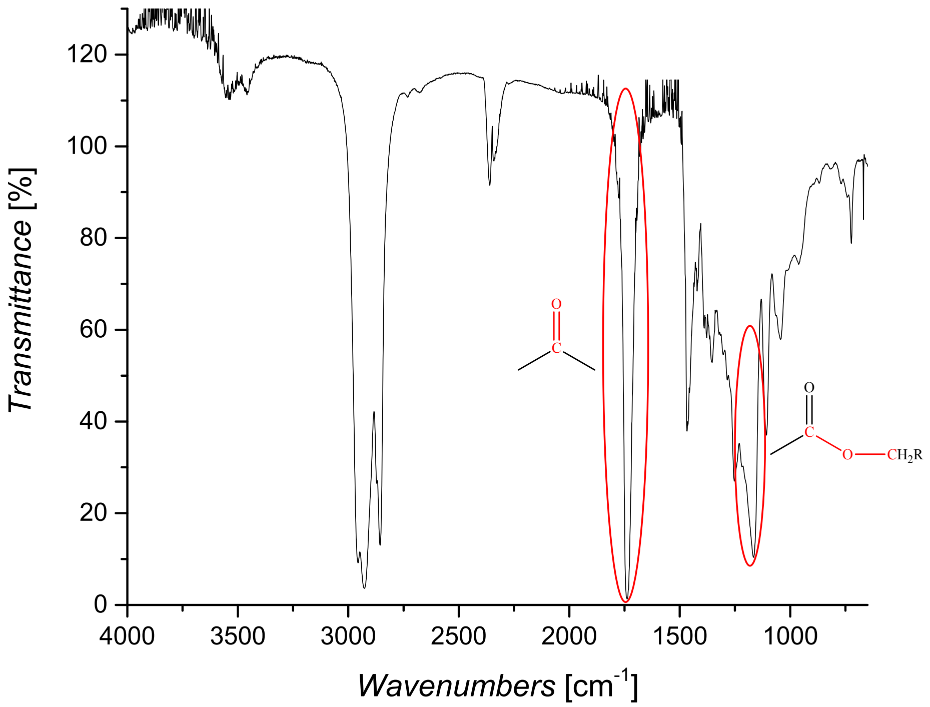

Figure S.5- a) ^{1}H-NMR and b) FT-IR spectra of 1,4-butanediol dipelargonate.

a)

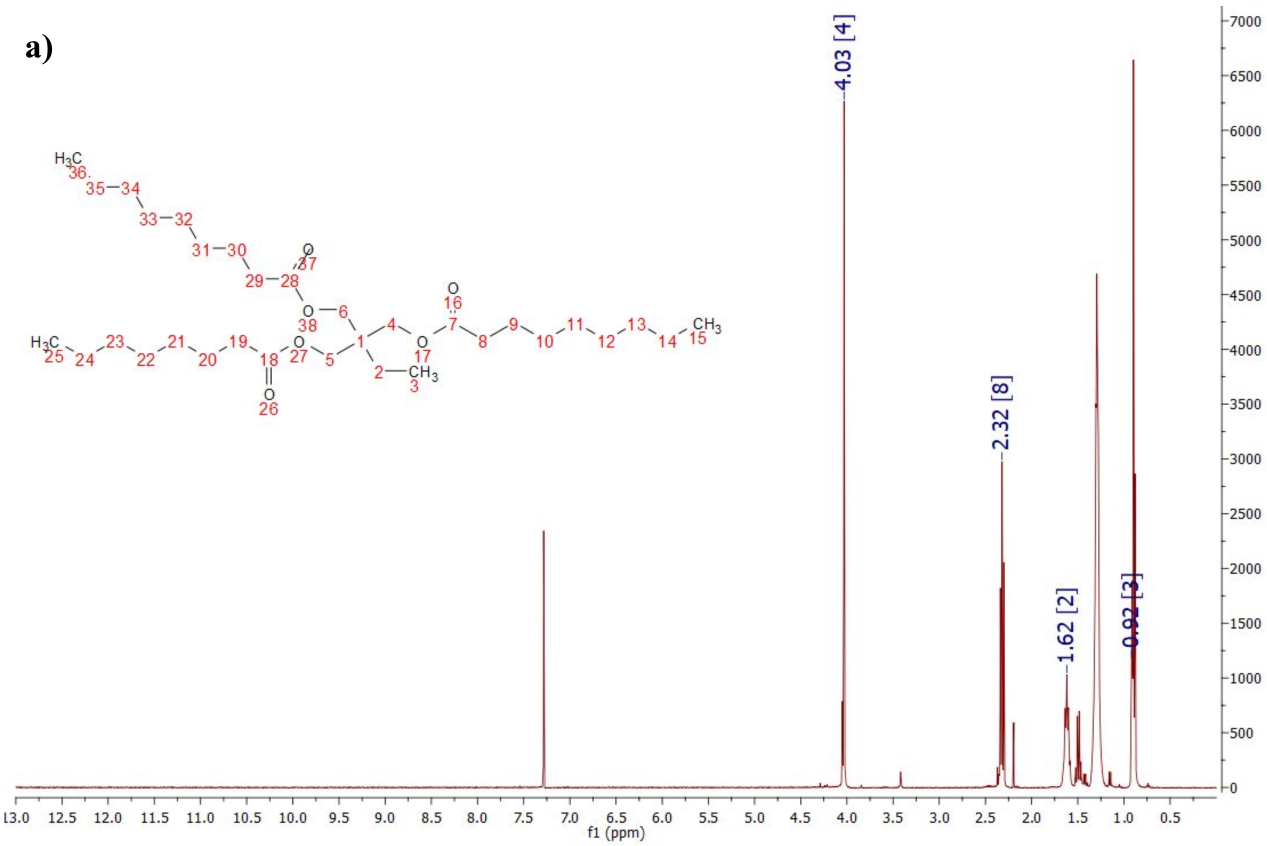

b)

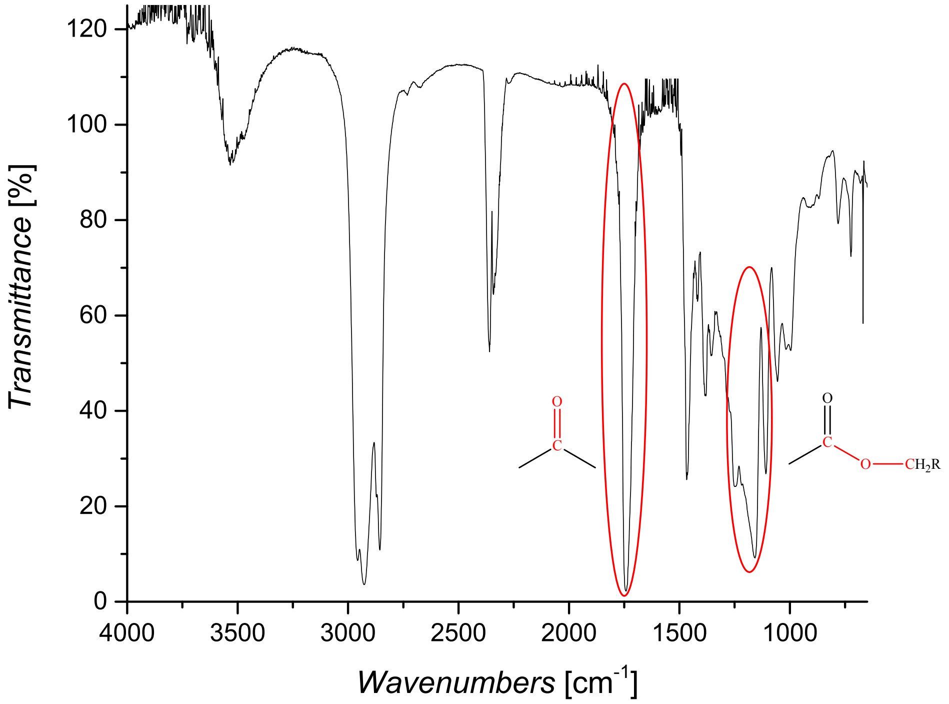

Figure S.6- a)^{1}H-NMR and b) FT-IR spectra of TMP tripelargonate.

a)

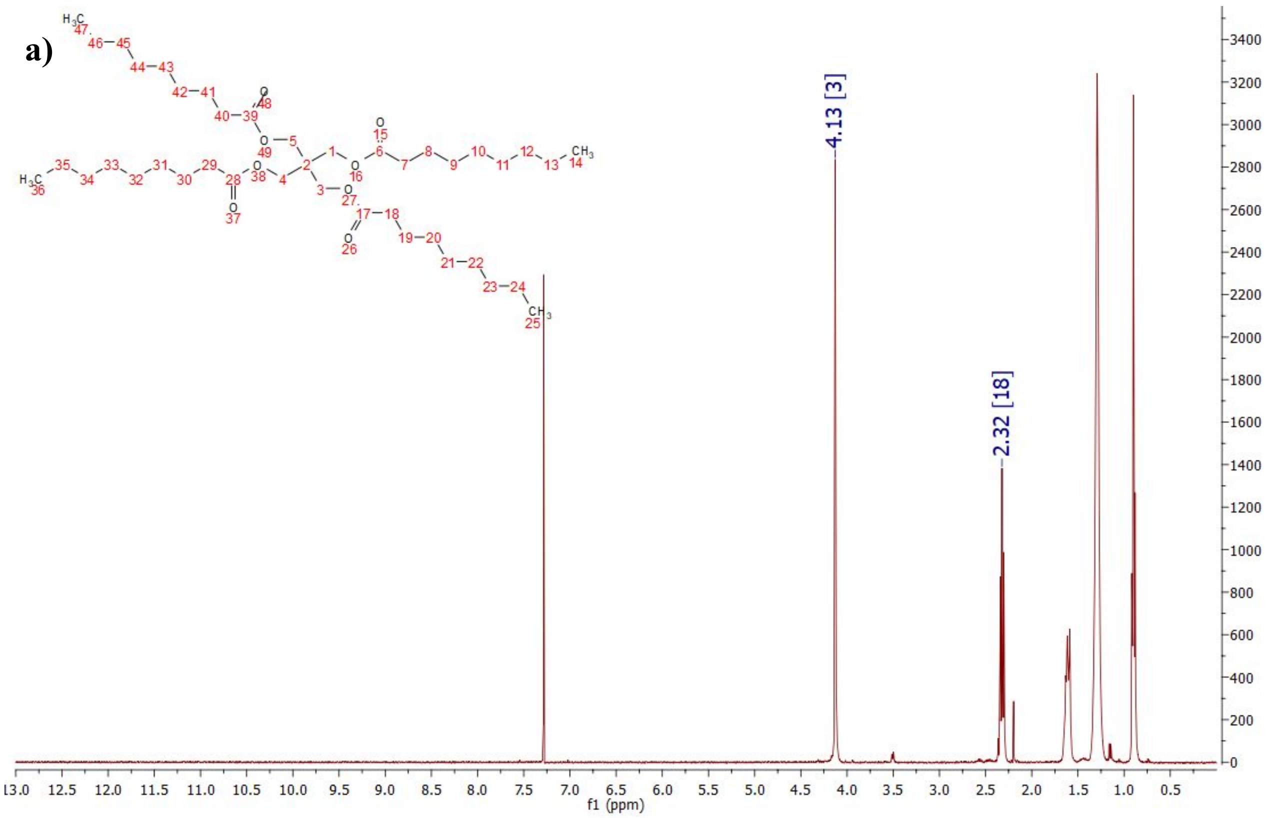

b)

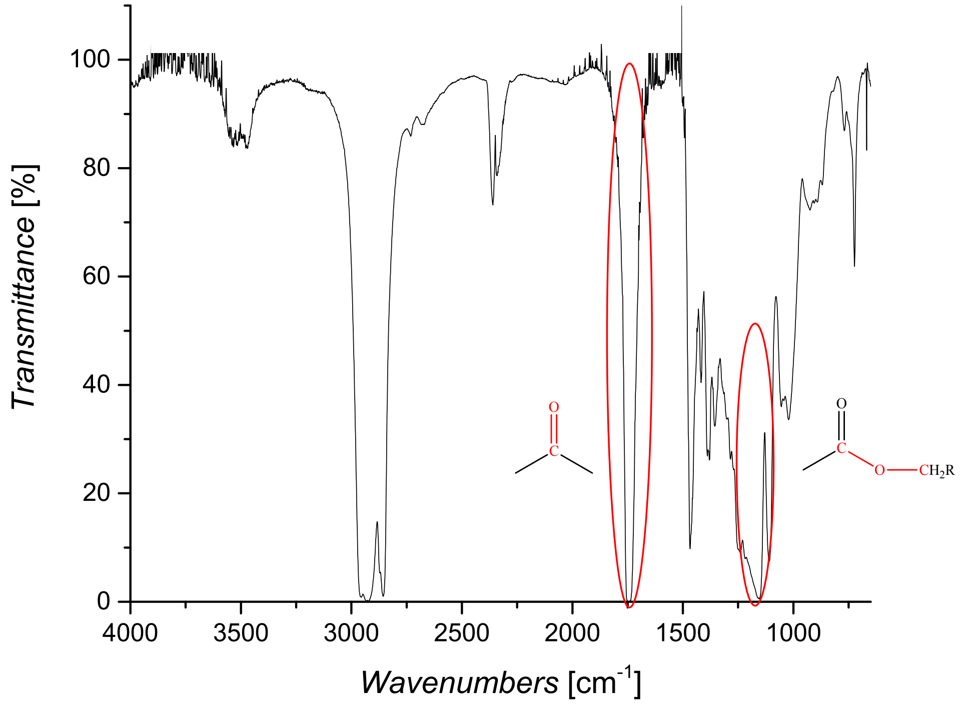

Figure S.7- a) ^{1}H-NMR and b) FT-IR spectra of pentaerythritol tetrapelargonate.

a)

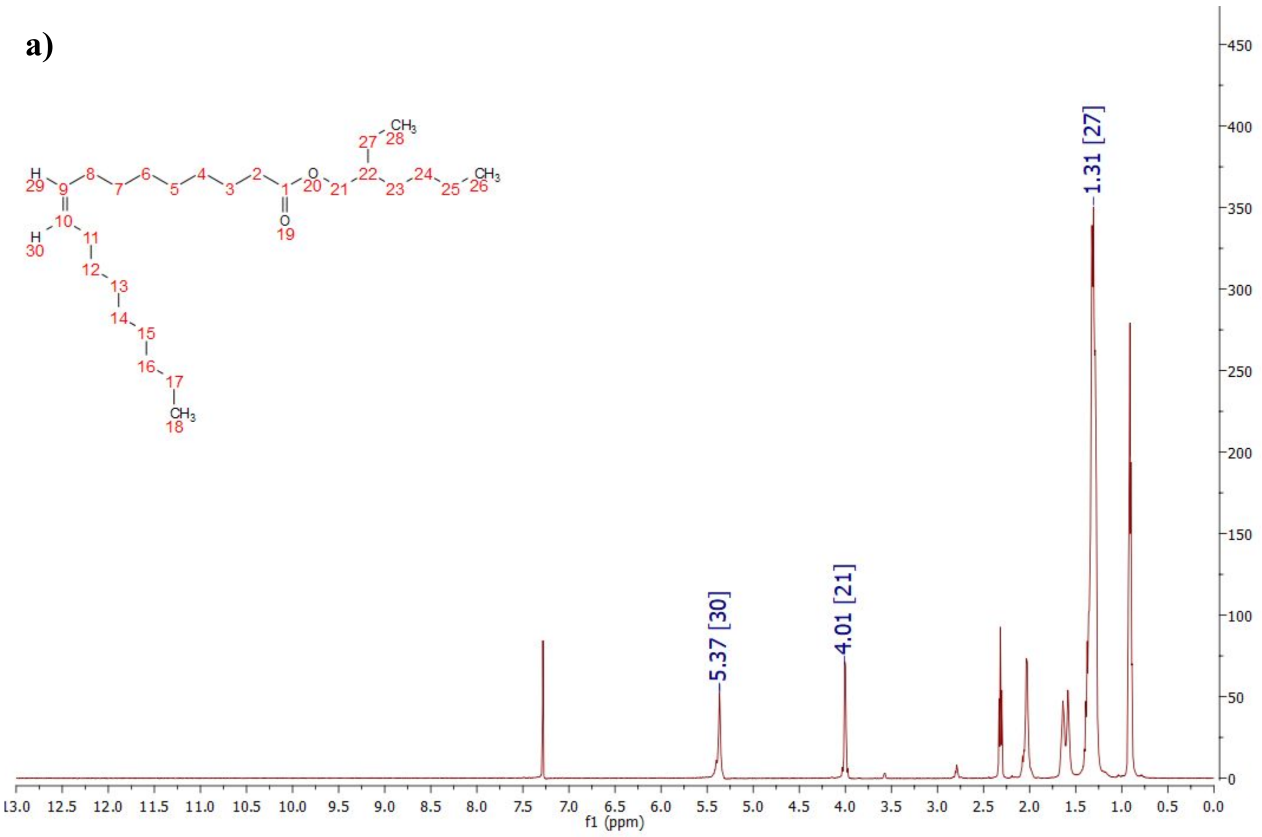

b)

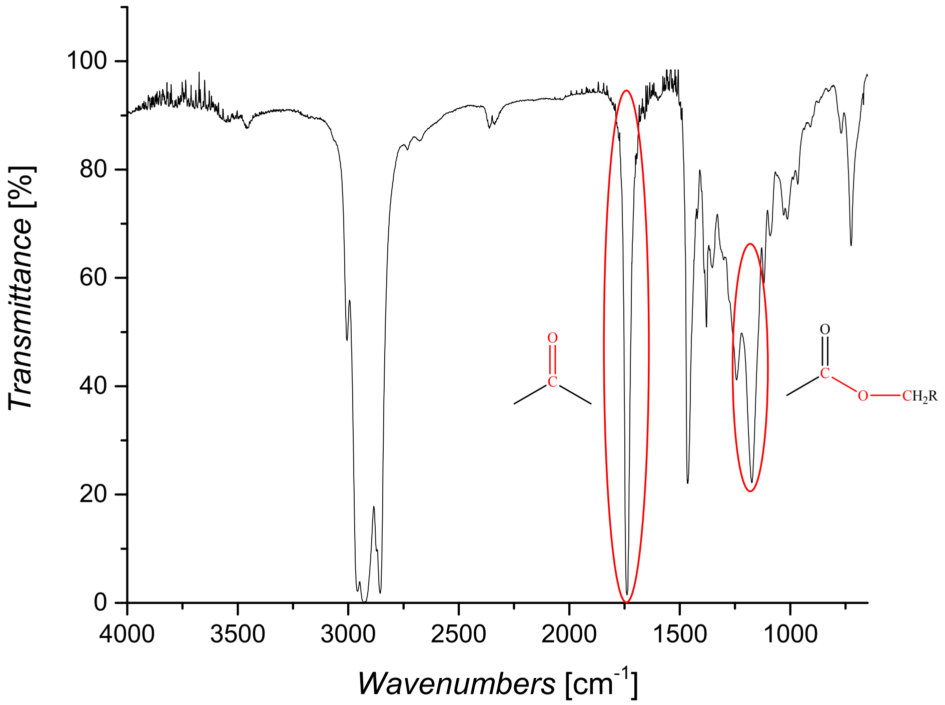

Figure S.8- a) ^{1}H-NMR and b) FT-IR spectra of 2-ethylhexyl oleate.

a)

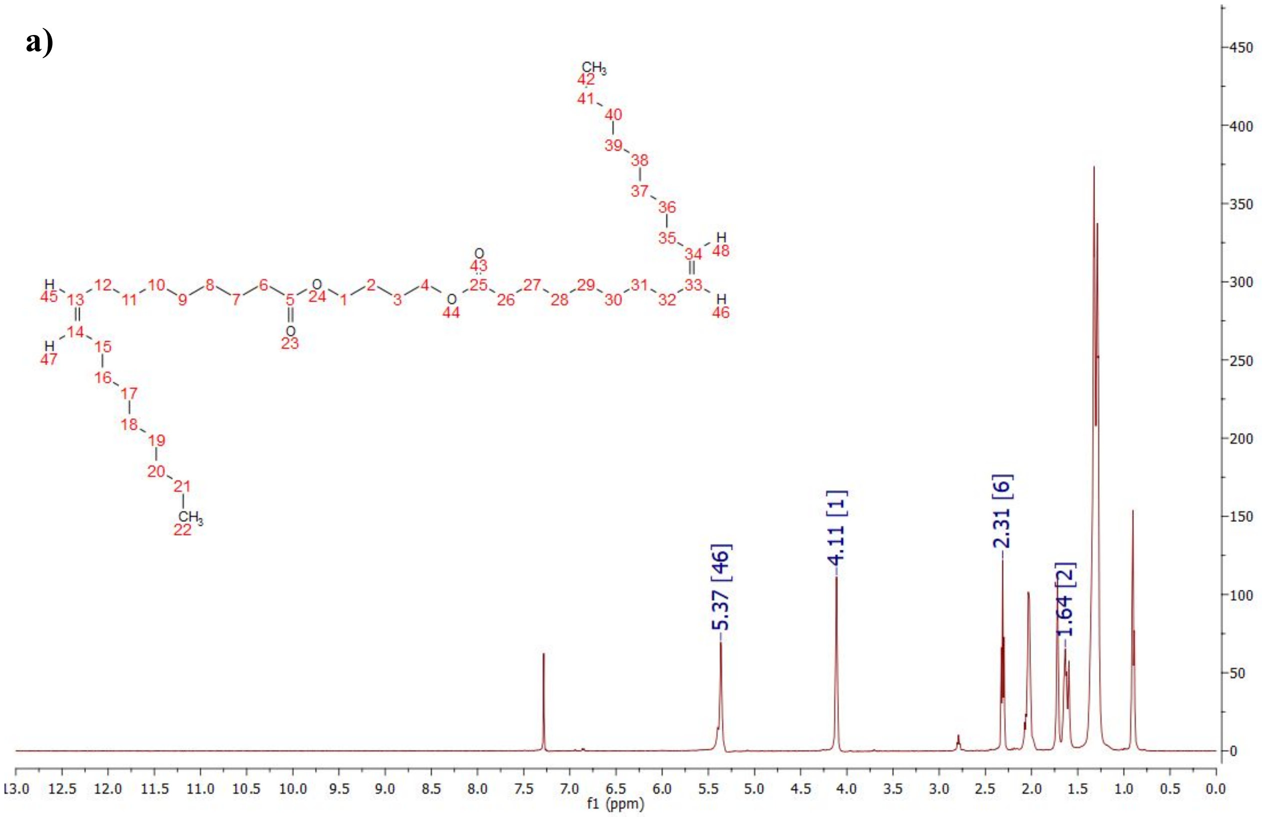

b)

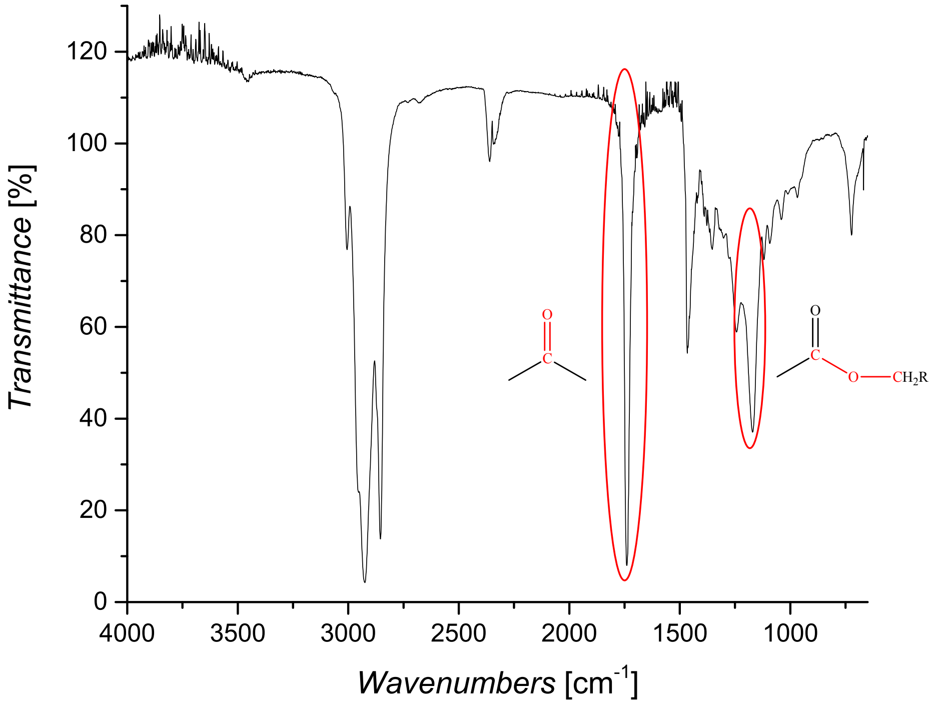

Figure S.9- a) ^{1}H-NMR and b) FT-IR spectra of 1,4-butanediol dioleate.

a)

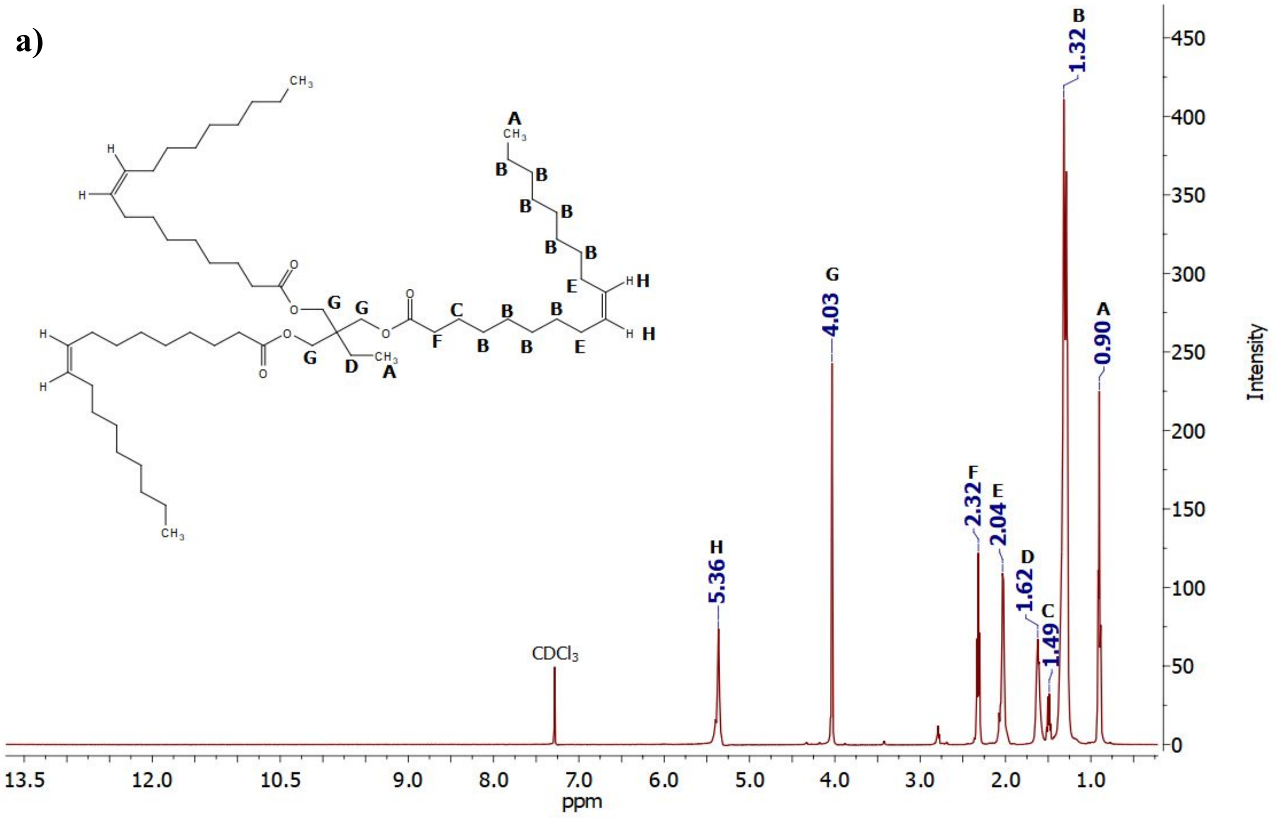

b)

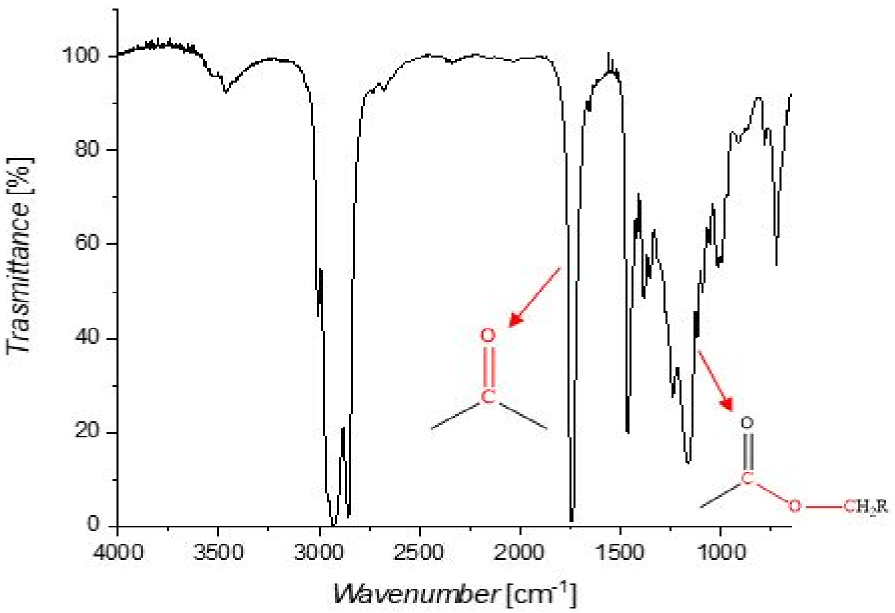

Figure S.10- a) ^{1}H-NMR and b) FT-IR spectra of TMP trioleate.

a)

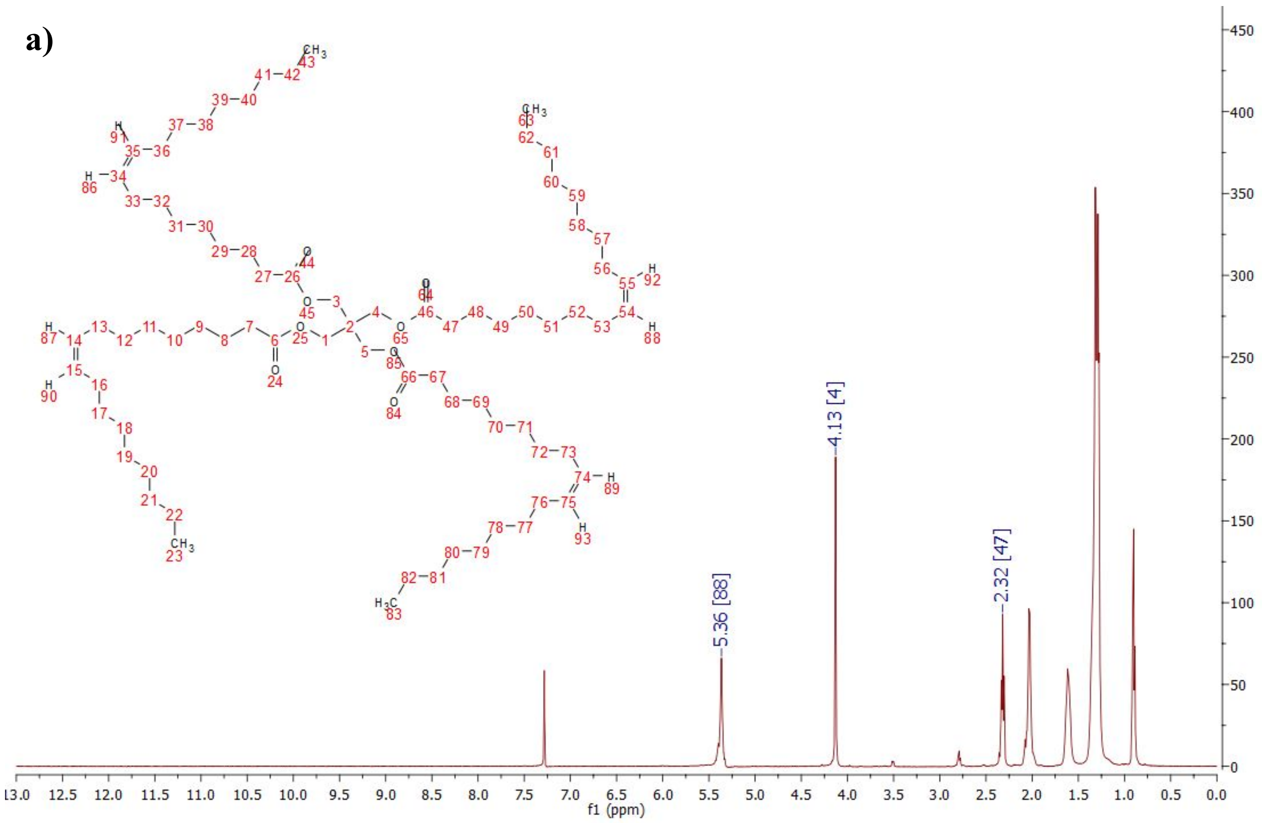

b)

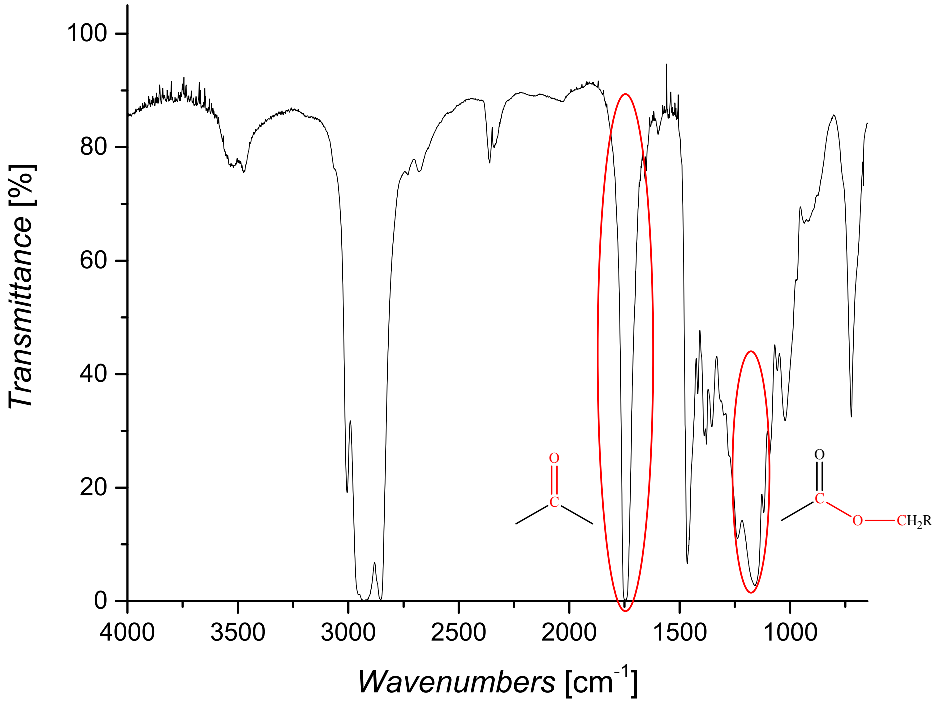

Figure S.11- a)^{1}H-NMR and b) FT-IR spectra of pentaerythritol tetraoleate.

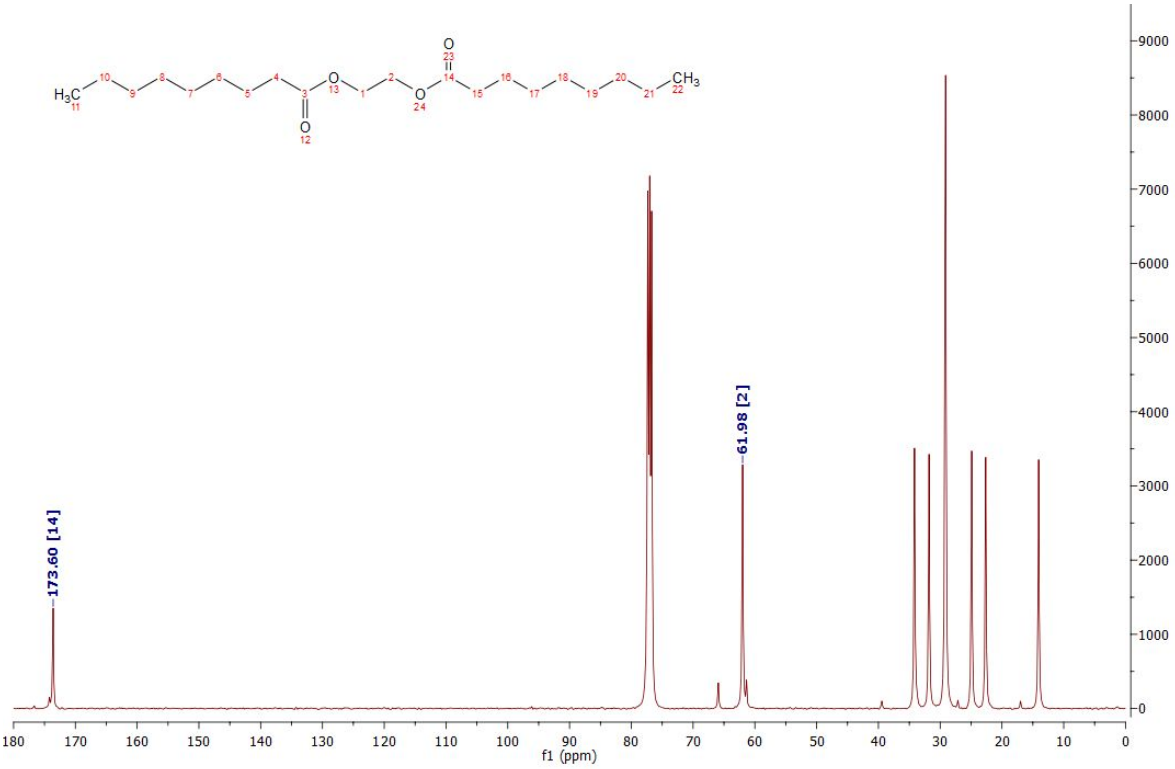

Figure S.12- ^{13}C-NMR spectrum of ethylene glycol dipelargonate.

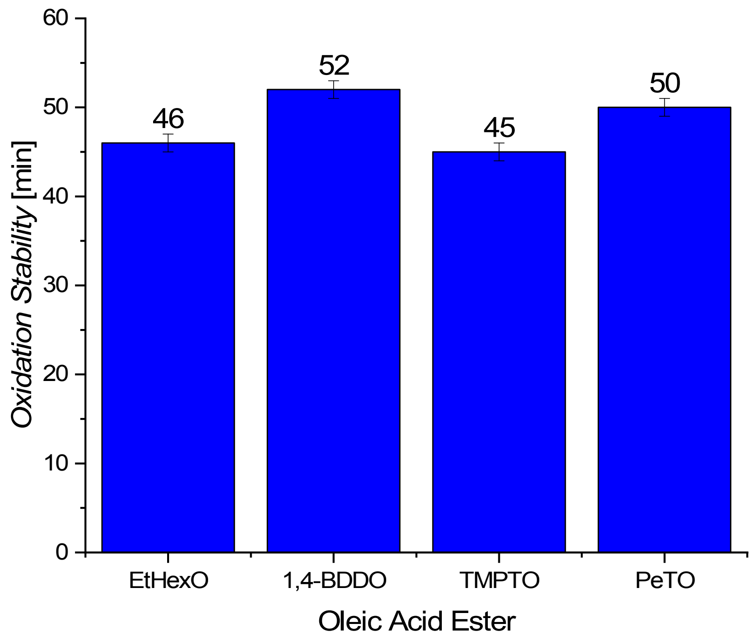

Figure S.13- Trend of the oxidation stabilities of oleates.

Determination of the purity of the fatty acid alkyl esters by ¹H-NMR

The purity of the synthetized fatty acid alkyl esters was evaluated by quantitative ^{1}H-NMR. The absence of signals related to by-products coming from side reactions allowed the definition of purity as equal to the yield of the specific ester. In particular, the triplet at 2.32 ppm attributed to the methylene vicinal to the carbonylic group (-CH_2CO-) of the fatty acid backbone was set as reference peak. Thus, the purity was calculated as in Equation S.1.

$$Purity\ \ [\%] = \frac{I_{FAAE}}{I_{FA}} \cdot 100 \qquad \text{(S.1)}$$

where I_{FAAE} is the integral values of the ester's peak (related to the to the methylene groups of the alcohol backbone bonded to the carboxylate groups of the fatty acid chains -CH_2OC(O)R and I_{FA} is the integral value of the reference peak.
